# A retrospective study on the impact of radiotherapy on the survival outcomes of small cell lung cancer patients based on the SEER database

**DOI:** 10.1038/s41598-024-65314-8

**Published:** 2024-07-05

**Authors:** Yao Chen, Ling Yao, Qingquan Chen, Yiming Hu, Xi Zhu, Rongrong Dai, Xiaoyang Chen, Yifu Zeng, Yong Zhu, Duanhong Song, Yixiang Zhang

**Affiliations:** 1 The Second Affiliated Hospital of Fujian Medical University, Quanzhou, 362000 Fujian Province China; 2 The School of Public Health, Fujian Medical University, Fuzhou, 350108 Fujian Province China; 3 College of Clinical Medicine for Obstetrics and Gynecology and Pediatrics, Fujian Children’s Hospital, Fujian Medical University, Fuzhou, 350014 China; 4 National Center for Chronic and Noncommunicable Disease Control and Prevention, Chinese Center for Disease Control and Prevention, Beijing, 100050 China; 5 Cyberspace Institute of Advanced Technology, Guangzhou University, Guangzhou, China; 6 Department of Thoracic Surgery, Fujian Medical University Union Hospital, Fuzhou, China

**Keywords:** Small cell lung cancer, Radiotherapy, Cancer-specific survival, Propensity score-matched analysis, SEER, Lung cancer, Cancer epidemiology, Epidemiology

## Abstract

Small cell lung cancer (SCLC) patients exhibit significant heterogeneity in tumor burden, physical condition, and responses to initial treatment. This diversity in treatment responses can result in varying treatment outcomes. The primary objective of this study was to explore the patient demographics associated with improved survival outcomes through radiotherapy. Based on the SEER database, we identified 42,824 SCLC patients enrolled between 2004 and 2015. These patients were stratified into radiotherapy (n = 20,360) and non-radiotherapy groups (n = 22,464). We controlled for confounding factors using propensity score matching (PSM) analysis. Subsequently, Kaplan–Meier (KM) analysis was employed to evaluate the impact of radiotherapy on patients’ overall survival (OS) and cancer-specific survival (CSS). Cancer-specific mortality was further analyzed using competitive risk models. Cox analysis was also conducted to examine additional variables potentially affecting the survival of SCLC patients. We identified a total of 42,824 eligible patients, and following PSM, 13,329 patients were successfully matched in both the radiotherapy and non-radiotherapy groups. The KM analysis showed that the median OS was 9 months in the radiotherapy group and 6 months in the non-radiotherapy group. The median CSS was 10 months in the radiotherapy group and 7 months in the non-radiotherapy group. The 5-year OS and 10-year OS rates were 6.2% versus 1.6% in the radiotherapy group and 2.6% versus 0.8% in the non-radiotherapy group (*P* < 0.001). Competitive risk analysis showed that cancer-specific mortality was significantly higher in the non-radiotherapy group than in the radiotherapy group (*P* < 0.001). Multivariate Cox analysis showed that the radiotherapy group (relative non-radiotherapy group) showed a significant positive effect on survival outcomes (OS: HR 0.658 95% CI [0.642, 0.675] *P* < 0.001; CSS: HR 0.662 95% CI [0.645, 0.679], *P* < 0.001). In addition, age, gender, race, primary tumor site, T stage, N stage, M stage, chemotherapy, and surgery were also considered as important predictors of SCLC outcome. The results of the subgroup analysis showed that the radiotherapy group showed a significant survival advantage regardless of age, sex, race, primary tumor site, M stage, chemotherapy, and surgery (*P* < 0.001). Radiotherapy may improve both OS and CSS in SCLC patients. Patients with SCLC may benefit from radiotherapy regardless of age, sex, race, primary tumor site, M stage, chemotherapy, and surgery.

## Introduction

According to the American Cancer Society (ACS), lung cancer is the second most common cancer and the leading cause of cancer deaths, with 238,340 new cases and 127,070 deaths in 2023^1^. Small cell lung cancer (SCLC) accounts for approximately 13% of all lung cancers, and it is a highly aggressive, poorly differentiated, high-grade neuroendocrine carcinoma^2^. Compared with non-small cell lung cancer (NSCLC), SCLC is characterized by a rapid multiplication time, early and extensive metastasis, and a poor prognosis, and its 5-year overall survival (OS) less than 10%^3,4^.

Radiotherapy is a common treatment modality for cancer that modulates the immune tumor microenvironment (TME) in various ways. On the one hand, it induces killing of tumor cells by causing DNA strand breaks^5^. On the other hand, it also activates the killing action of immune cells to cause damage to target tumor cells through adequate antigenic and co-stimulation provided by antigen-presenting cells (APCs) and chemokine secretion^6^. According to the Veterans Administration Lung Study Group (VALSG) two-classification methodology classifies small cell lung cancer into limited stage (LS) and extensive stage (ES)^7^. The mainstay of treatment for SCLC is chemotherapy, but it is also very sensitive to radiation therapy. LS-SCLC is a potentially curable disease with a long-term survival rate of 20–25% when treated with platinum-based chemotherapy plus simultaneous thoracic radiotherapy^8^. Hyperfractionated (twice-daily) chest radiotherapy and prophylactic cranial irradiation (PCI) may improve survival in selected LS-SCLC patients^8^. And for ES-SCLC patients, radiotherapy has a place in the mix. PCI reduces the risk of brain metastases and significantly improves overall survival, so cisplatin (or carboplatin)-etoposide followed by PCI has become the standard of care for responding patients^9^. Although there is insufficient evidence to recommend that the addition of chest radiotherapy to standard chemotherapy provides a survival benefit for ES-SCLC, chest radiotherapy reduces the chance of recurrence^10^. A phase 3 randomized controlled trial found that overall survival at 1 year was not significantly different between groups: 33% (95% CI 27–39) for the thoracic radiotherapy group versus 28% (95% CI 22–34) for the control group (hazard ratio [HR] 0.84, 95% CI 0.69–1.01; *p* = 0.066)^11^. However, in a secondary analysis, 2-year overall survival was 13% (95% CI 9–19) versus 3% (95% CI 2–8; *p* = 0.004)^11^. The thoracic radiotherapy group was less likely to have disease progression (HR 0.73, 95% CI 0.61–0.87; *p* = 0.001)^11^. Thus in patients with residual intrathoracic disease and low-volume extrathoracic disease, who may be at greater risk of intrathoracic progression, radiotherapy may be considered for these subgroups of patients^10^.

SCLC patients have high heterogeneity in terms of tumor load, physical status, and response to initial treatment. The same treatment in different subgroups may lead to different outcomes. In this study, SCLC with complete data from 2004 to 2015 were extracted using the Surveillance, Epidemiology, and End Results (SEER) database. analyses were performed to assess the effect of radiotherapy on OS and CSS in SCLC patients.

## Materials and methods

### Ethics approval and consent to participate

The SEER database provides publicly available data for this study, which means that obtaining informed consent from participants or ethical approval from an institutional review board is not necessary. We obtained access to the 1979–2019 SEER Research Data File by signing a Data-Use Agreement that outlines the terms and conditions for access.

### Data sources

The primary objective of this study was to investigate the correlation between radiotherapy and the survival prognosis of SCLC patients. All data are based on the SEER database, which was established in 1973 to collect information on cancer incidence and survival in the United States. It comprehensively covers data from 17 population-based cancer registries, effectively representing approximately 28% of the current U.S. population^12^.

### Study population

**Figure 1:**
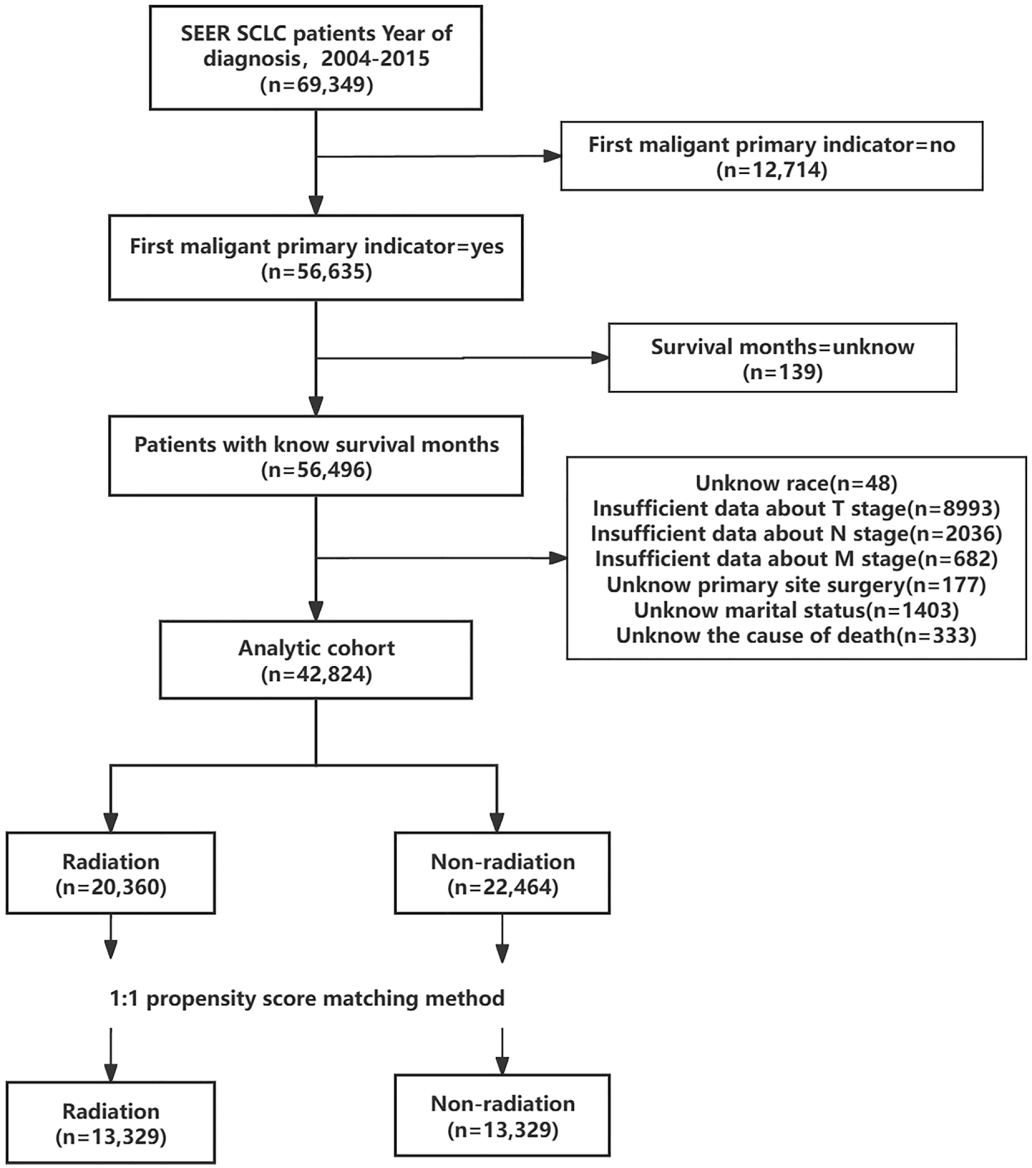
Flowchart illustrating the patient selection process based on inclusion and exclusion criteria.

The study population consisted of patients diagnosed with primary SCLC from 2004 to 2015. These patient records were extracted from SEER*Stat software (version 8.4.2). Inclusion criteria were as follows: (1) Diagnosis within the years 2004–2015; (2) Primary site identified as "lung and bronchus" (according to ICD-O-3/WHO 2008); and (3) Histologic classification coded as 8002/3, 8041/3, 8042/3, 8043/3, 8044/3, or 8045/3. Exclusion criteria included: (1) First malignancy primary indicator = no (n = 12,714); (2) Unknown survival time (n = 139); (3) Unknown race (n = 48); (4) TX (n = 8993); (5) NX (n = 2036); (6) MX (n = 682); (7) Unavailable surgical information (n = 177); (8) Unknown marital status (n = 1403); and (9) Unclear cause of death (n = 333) (Fig. 1).

### Covariates

Baseline clinical characteristics were collected, including age, sex, race, primary site, laterality, TNM stage, chemotherapy, surgery, marital status, and median household income.

### Statistical analyses

Categorical variables were compared using the chi-square. Potential biases between the radiotherapy and non-radiotherapy groups were mitigated through propensity score matching (PSM) analysis. The matching tolerance (caliper) was set to 0.001, and a 1:1 nearest neighbor matching strategy was implemented. OS is the time from tumour diagnosis to any cause of death or the time of last follow-up. The CSS only represents the status in which the patient died from lung cancer alone. In the competing risk model, death was defined as two groups: lung cancer death and death from other diseases. Kaplan–Meier (KM) survival analysis, accompanied by the log-rank test, was employed to compare overall survival (OS) and cancer-specific survival (CSS) among patients who received radiotherapy and those who did not. Cancer-specific mortality was further analyzed using competitive risk models, and group differences were estimated by the Gray test, which was somewhat superior to the Kaplan–Meier method and Cox regression models.

All statistical analyses were executed using R version 4.3.1. The R package "MatchIt" was utilized for the propensity score matching. All statistical tests were two-tailed, and a significance level of *P* < 0.05 was deemed statistically significant.

## Results

### Study cohort characteristics

The SEER database furnished data for a total of 69,349 SCLC patients, enabling their inclusion in our study. Following a comprehensive screening process, 42,824 individuals were identified as suitable candidates, as depicted in Fig. 1. Among these eligible patients, 20,360 (47.54%) underwent radiotherapy, while 22,464 (52.46%) did not. In the radiotherapy group, 87.8% of the patients received concurrent chemotherapy, compared to 56.5% in the non-radiotherapy group. Additionally, 2.6% of patients in the radiotherapy group underwent surgical treatment during the same period, whereas it was 3.3% in the non-radiotherapy group. More detailed demographic information can be found in Table 1. Moreover, after initial comparisons were conducted, significant disparities were observed between the radiotherapy and non-radiotherapy groups concerning age, race, primary site, laterality, TNM stage, surgical status, chemotherapy status, marital status, and median household income, all with *P* ≤ 0.001 (Table 1).

**Table 1:** Baseline characteristics of SCLC patients based on radiotherapy status.

Characteristic	Radiation (n = 20,360)	Non-radiation (n = 22,464)	*P*
Age (%)			
< 70	13,980 (68.7)	11,615 (51.7)	< 0.001
≥ 70	6380 (31.3)	10,849 (48.3)	
Gender (%)			
Female	10,306 (50.6)	11,039 (49.1)	0.002
Male	10,054 (49.4)	11,425 (50.9)	
Race (%)			
Black	1872 (9.2)	1768 (7.9)	< 0.001
Other	899 (4.4)	907 (4.0)	
White	17,589 (86.4)	19,789 (88.1)	
Primary Site (%)			
Main bronchus	2501 (12.3)	2704 (12.0)	< 0.001
Upper lobe, lung	10,434 (51.2)	9943 (44.3)	
Middle lobe, lung	826 (4.1)	908 (4.0)	
Lower lobe, lung	3919 (19.2)	4658 (20.7)	
Overlapping lesion of lung	290 (1.4)	398 (1.8)	
Lung, NOS	2390 (11.7)	3853 (17.2)	
Laterality (%)			
Bilateral, single primary	103 (0.5)	284 (1.3)	< 0.001
Left—origin of primary	8167 (40.1)	9210 (41.0)	
Not a paired site	49 (0.2)	49 (0.2)	
Only one side—side unspecified	42 (0.2)	75 (0.3)	
Paired site, but no information concerning laterality	342 (1.7)	647 (2.9)	
Right—origin of primary	11,657 (57.3)	12,199 (54.3)	
T (%)			
T0	231 (1.1)	265 (1.2)	< 0.001
T1	2716 (13.3)	2316 (10.3)	
T2	5684 (27.9)	5442 (24.2)	
T3	1052 (5.2)	898 (4.0)	
T4	10,677 (52.4)	13,543 (60.3)	
N (%)			
N0	3121 (15.3)	3629 (16.2)	< 0.001
N1	1615 (7.9)	1544 (6.9)	
N2	11,598 (57.0)	12,899 (57.4)	
N3	4026 (19.8)	4392 (19.6)	
M (%)			
M0	9722 (47.8)	6416 (28.6)	< 0.001
M1	10,638 (52.2)	16,048 (71.4)	
Surgery (%)			
No	19,831 (97.4)	21,720 (96.7)	< 0.001
Yes	529 (2.6)	744 (3.3)	
Chemotherapy (%)			
No/Unknown	2474 (12.2)	9773 (43.5)	< 0.001
Yes	17,886 (87.8)	12,691 (56.5)	
Marital status (%)			
Divorced/Separated	3466 (17.0)	3534 (15.7)	< 0.001
Married	10,978 (53.9)	10,871 (48.4)	
Single	2845 (14.0)	3196 (14.2)	
Widowed	3071 (15.1)	4863 (21.6)	
Median household income (%)			
< 35,000	693 (3.4)	749 (3.3)	< 0.001
> 75,000	4531 (22.3)	4848 (21.6)	
35,000–54,999	6859 (33.7)	7117 (31.7)	
55,000–74,999	8277 (40.7)	9750 (43.4)	

### Propensity score matching (PSM)

**Figure 2:**
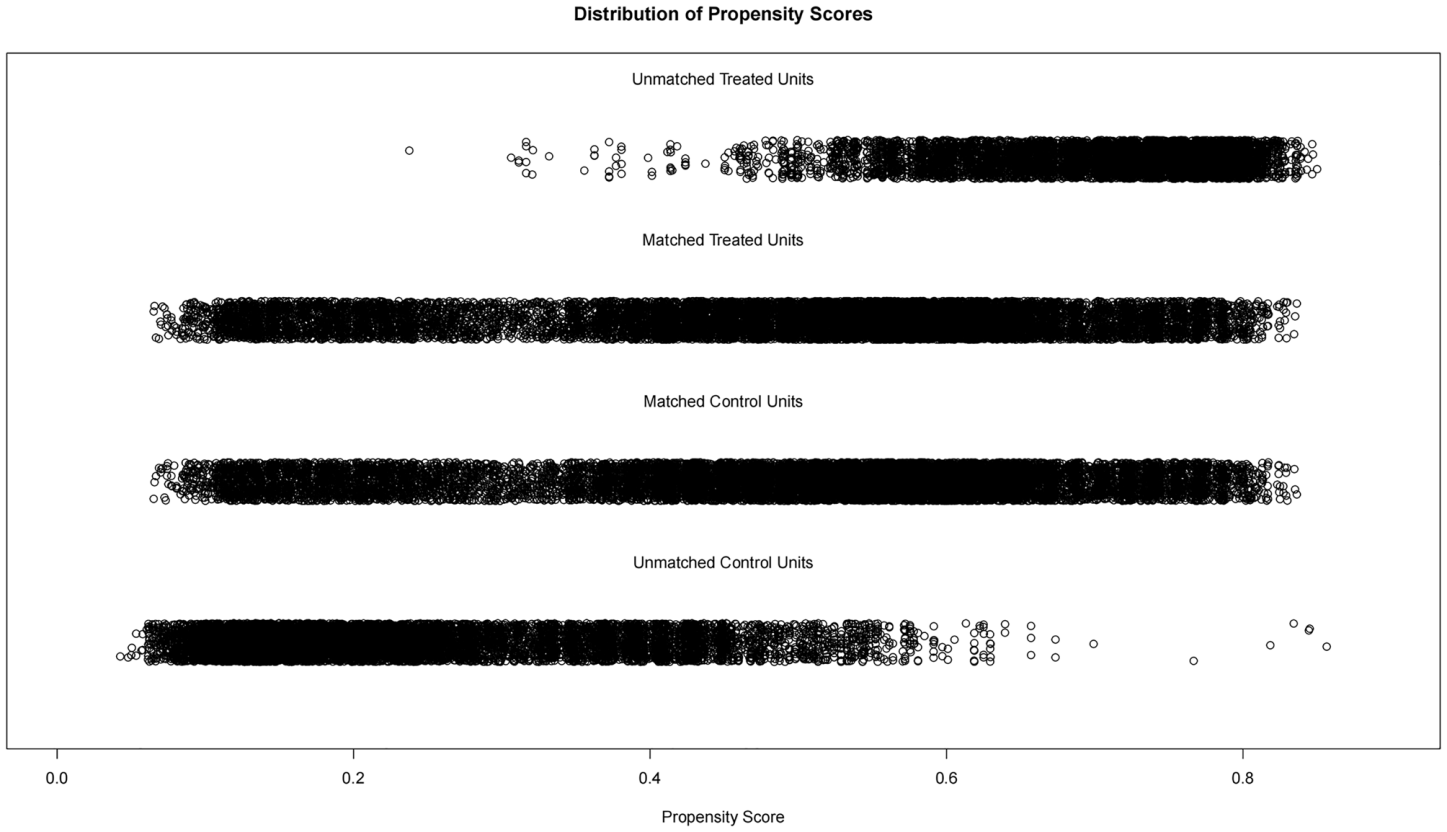
Propensity score matching for radiotherapy and non-radiotherapy groups.

To mitigate potential confounding variables such as age, gender, and race between the radiotherapy and non-radiotherapy groups, we employed a 1:1 propensity score matching method, resulting in a total of 13,329 patients matched between the two groups. The distribution of covariates between the groups before and after matching was compared (Fig. 2). Post-matching, most covariates exhibited *P* > 0.05, indicating negligible differences between the two groups (Table 2, Fig. 2).

**Table 2:** Baseline characteristics of patients with SCLC based on radiotherapy status after propensity score matching.

Characteristic	Radiation (n = 13,329)	Non-radiation (n = 13,329)	*P*
Age (%)			
< 70	8192 (61.5)	8309 (62.3)	0.143
≥ 70	5137 (38.5)	5020 (37.7)	
Gender(%)			
Female	6471 (48.5)	6511 (48.8)	0.633
Male	6858 (51.5)	6818 (51.2)	
Race (%)			
Black	1187 (8.9)	1080 (8.1)	0.001
Other	624 (4.7)	527 (4.0)	
White	11,518 (86.4)	11,722 (87.9)	
Primary site (%)			
Main bronchus	1665 (12.5)	1603 (12.0)	0.011
Upper lobe, lung	6210 (46.6)	6517 (48.9)	
Middle lobe, lung	539 (4.0)	522 (3.9)	
Lower lobe, lung	2784 (20.9)	2653 (19.9)	
Overlapping lesion of lung	226 (1.7)	202 (1.5)	
Lung, NOS	1905 (14.3)	1832 (13.7)	
Laterality (%)			
Bilateral, single primary	100 (0.8)	86 (0.6)	0.609
Left—origin of primary	5473 (41.1)	5483 (41.1)	
Not a paired site	38 (0.3)	35 (0.3)	
Only one side—side unspecified	38 (0.3)	31 (0.2)	
Paired site, but no information concerning laterality	302 (2.3)	272 (2.0)	
Right—origin of primary	7378 (55.4)	7422(55.7)	
T			
T0	169 (1.3)	145 (1.1)	0.062
T1	1504 (11.3)	1503 (11.3)	
T2	3302 (24.8)	3422 (25.7)	
T3	619 (4.6)	543 (4.1)	
T4	7735 (58.0)	7716 (57.9)	
N			
N0	2031 (15.2)	1964 (14.7)	0.232
N1	962 (7.2)	910 (6.8)	
N2	7480 (56.1)	7632 (57.3)	
N3	2856 (21.4)	2823 (21.2)	
M			
M0	3996 (30.0)	4124 (30.9)	0.091
M1	9333 (70.0)	9205 (69.1)	
Surgery (%)			
No	12,886 (96.7)	12,975 (97.3)	0.002
Yes	443 (3.3)	354 (2.7)	
Chemotherapy (%)			
No/Unknown	2464 (18.5)	2641 (19.8)	0.006
Yes	10,865 (81.5)	10,688 (80.2)	
Marital status (%)			
Divorced/Separated	2239 (16.8)	2195 (16.5)	0.21
Married	6832 (51.3)	7003 (52.5)	
Single	1866 (14.0)	1821 (13.7)	
Widowed	2392 (17.9)	2310 (17.3)	
Median household income (%)			
< 35,000	468 (3.5)	417 (3.1)	0.094
> 75,000	3005 (22.5)	2919 (21.9)	
35,000–54,999	4411 (33.1)	4393 (33.0)	
55,000–74,999	5445 (40.9)	5600 (42.0)	

### Comparison of survival curves between radiotherapy and non-radiotherapy groups

**Figure 3:**
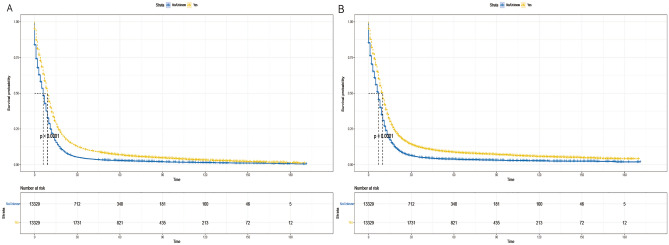
Kaplan–Meier survival curves for patients between radiation and non-radiotherapy groups. (**A**) overall survival, (**B**) cancer-specific survival.

The Kaplan–Meier analysis showed that the median OS was 9 months in the radiotherapy group and 6 months in the non-radiotherapy group, and the median CSS was 10 months in the radiotherapy group and 7 months in the non-radiotherapy group. The 5-year and 10-year OS rates were 6.2% versus 1.6% in the radiotherapy group, and 2.6% versus 0.8% in the non-radiotherapy group (*P* < 0.001) (Fig. 3).

### Analysis of competing risk between radiotherapy versus non-radiotherapy groups

**Figure 4:**
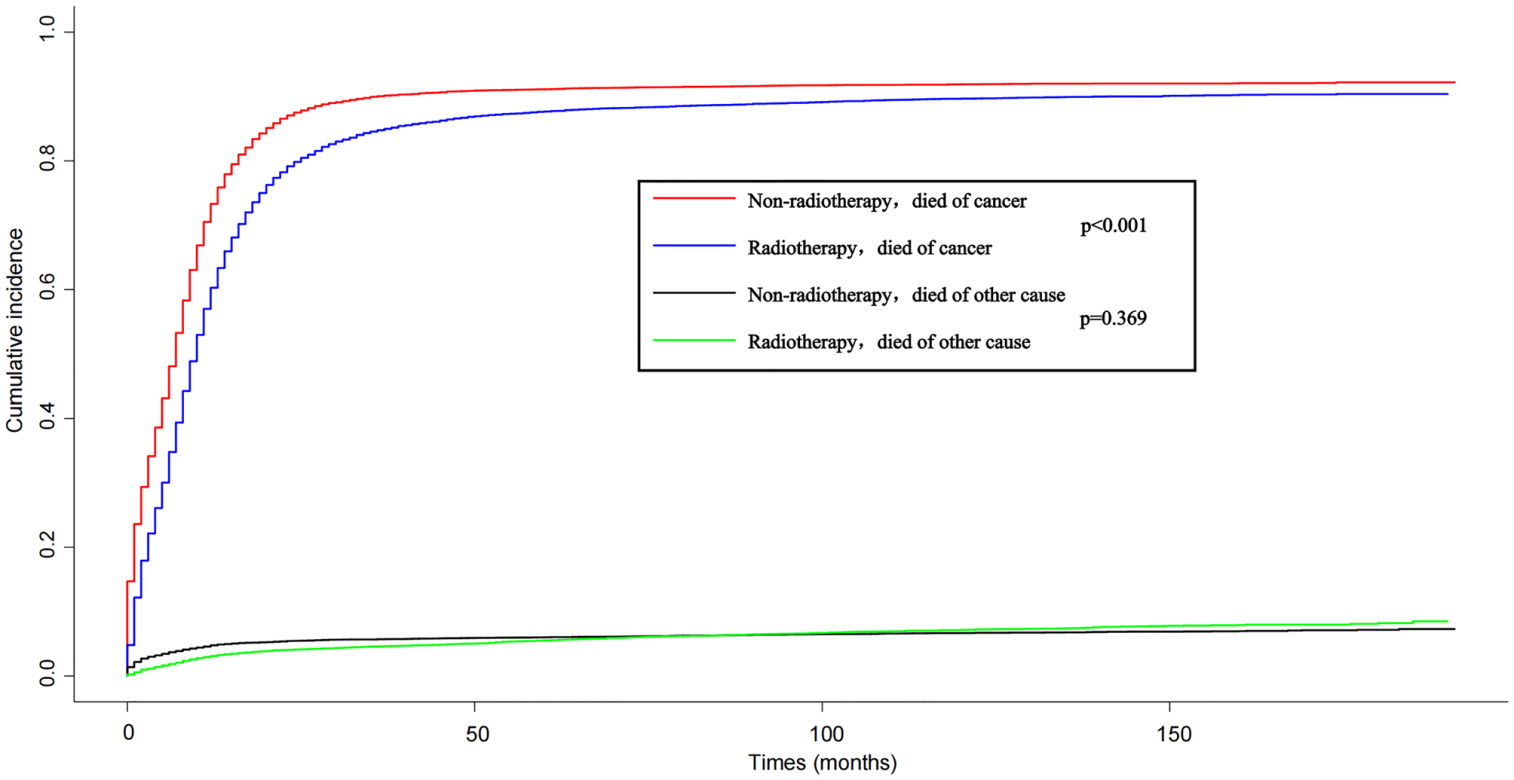
Competitive risk curves for cancer-specific survival in patients with SCLC.

The cumulative incidence function (CIF) is utilized to mitigate the influence of competing risks on death from other causes. It estimates the cumulative incidence of SCLC deaths alongside cancer-specific deaths, with deaths not attributed to SCLC regarded as competing events. In the competing risk model (refer to Fig. 4), the rate of cancer-specific mortality was notably higher in the non-radiotherapy group compared to the radiotherapy group (*P* < 0.001)(Fig. 4).

### Impact of radiotherapy on survival in patients with SCLC

In the univariate Cox regression analysis, all baseline characteristics emerged as significant predictors of OS and CSS in SCLC patients, except for median household income and marital status (Table 3). Radiotherapy (RT) was associated with improvements in both OS (HR 0.674, 95% CI [0.658, 0.691], *P* < 0.001) and CSS (HR 0.661, 95% CI [0.644, 0.678], *P* < 0.001). Upon further Cox regression analysis of relevant variables, all factors maintained independent significance in predicting OS/CSS, except for median family income, marital status, and laterality. Moreover, the radiotherapy group, relative to the non-radiotherapy group, exhibited a significant positive impact on survival outcomes (OS: HR 0.658, 95% CI [0.642, 0.675], *P* < 0.001; CSS: HR 0.662, 95% CI [0.645, 0.679], *P* < 0.001; see Table 3). Remarkably, patients aged under 70 years, female, of Black ethnicity, with primary lung tumors, T0 stage, N0 stage, who underwent surgery, exhibited M0 stage, and received chemotherapy were more likely to experience improvements in OS and CSS compared to their respective control groups (Table 3).

**Table 3:** Univariate and multivariate analysis of the effect of radiotherapy status on survival outcomes in SCLC patients.

Variables	OS				CSS			
	Univariate analysis		Multivariate analysis		Univariate analysis		Multivariate analysis	
	HR (95% CI)	*P*	HR (95% CI)	*P*	HR (95% CI)	*P*	HR (95% CI)	*P*
Age								
< 70	1		1		1		1	
≥ 70	1.212 [1.182, 1.242]	< 0.001	1.318 [1.284, 1.352]	< 0.001	1.166 [1.136, 1.197]	< 0.001	1.281 [1.247, 1.315]	< 0.001
Sex								
Female	1		1		1		1	
Male	1.190 [1.161, 1.220]	< 0.001	1.171 [1.143, 1.200]	< 0.001	1.192 [1.162, 1.223]	< 0.001	1.168 [1.139, 1.199]	< 0.001
Race								
Black	1		1		1		1	
Other	1.036 [0.964, 1.114]	0.333	1.004 [0.934, 1.080]	0.908	1.043 [0.968, 1.125]	0.269	1.014 [0.940, 1.093]	0.72
White	1.104 [1.057, 1.154]	< 0.001	1.106 [1.058, 1.155]	< 0.001	1.121 [1.070, 1.173]	< 0.001	1.121 [1.071, 1.174]	< 0.001
Primary site								
Main bronchus	1		1		1		1	
Upper lobe, lung	0.930 [0.895, 0.967]	< 0.001	0.949 [0.912, 0.987]	0.01	0.918 [0.882, 0.955]	< 0.001	0.946 [0.908, 0.985]	0.007
Middle lobe, lung	0.839 [0.782, 0.901]	< 0.001	0.937 [0.872, 1.006]	0.074	0.820 [0.762, 0.883]	< 0.001	0.925 [0.858, 0.996]	0.04
Lower lobe, lung	0.915 [0.876, 0.957]	< 0.001	1.009 [0.965, 1.055]	0.697	0.896 [0.856, 0.938]	< 0.001	1.002 [0.956, 1.049]	0.947
Overlapping lesion of lung	1.013 [0.915, 1.121]	0.807	0.977 [0.882, 1.082]	0.651	0.989 [0.889, 1.099]	0.831	0.952 [0.856, 1.058]	0.361
Lung, NOS	0.995 [0.949, 1.044]	0.838	1.013 [0.964, 1.066]	0.606	0.982 [0.935, 1.032]	0.476	1.008 [0.957, 1.062]	0.762
Laterality								
Bilateral, single primary	1		1		1		1	
Left—origin of primary	0.712 [0.616, 0.824]	< 0.001	0.939 [0.809, 1.089]	0.404	0.698 [0.601, 0.811]	< 0.001	0.935 [0.803, 1.089]	0.388
Not a paired site	0.670 [0.506, 0.886]	0.005	0.786 [0.592, 1.043]	0.095	0.698 [0.525, 0.928]	0.013	0.824 [0.618, 1.100]	0.189
Only one side—side unspecified	0.550 [0.413, 0.733]	< 0.001	0.807 [0.605, 1.077]	0.146	0.511 [0.376, 0.693]	< 0.001	0.771 [0.567, 1.048]	0.097
Paired site, but no information concerning laterality	0.714 [0.604, 0.844]	< 0.001	0.895 [0.753, 1.064]	0.208	0.685 [0.577, 0.815]	< 0.001	0.881 [0.737, 1.053]	0.162
Right—origin of primary	0.719 [0.621, 0.831]	< 0.001	0.935 [0.806, 1.085]	0.377	0.705 [0.607, 0.819]	< 0.001	0.933 [0.801, 1.087]	0.373
T								
T0	1		1		1		1	
T1	0.944 [0.837, 1.064]	0.342	1.064 [0.929, 1.219]	0.369	0.964 [0.848, 1.096]	0.579	1.090 [0.944, 1.260]	0.241
T2	1.276 [1.136, 1.434]	< 0.001	1.338 [1.172, 1.528]	< 0.001	1.334 [1.178, 1.512]	< 0.001	1.391 [1.208, 1.601]	< 0.001
T3	1.400 [1.232, 1.591]	< 0.001	1.452 [1.259, 1.673]	< 0.001	1.485 [1.296, 1.702]	< 0.001	1.525 [1.312, 1.773]	< 0.001
T4	1.505 [1.341, 1.689]	< 0.001	1.511 [1.327, 1.721]	< 0.001	1.602 [1.416, 1.812]	< 0.001	1.584 [1.379, 1.820]	< 0.001
N								
N0	1		1		1		1	
N1	1.130 [1.068, 1.195]	< 0.001	1.115 [1.054, 1.180]	< 0.001	1.159 [1.092, 1.230]	< 0.001	1.134 [1.068, 1.203]	< 0.001
N2	1.449 [1.397, 1.502]	< 0.001	1.309 [1.261, 1.359]	< 0.001	1.519 [1.462, 1.578]	< 0.001	1.350 [1.298, 1.405]	< 0.001
N3	1.460 [1.400, 1.522]	< 0.001	1.297 [1.241, 1.355]	< 0.001	1.535 [1.469, 1.604]	< 0.001	1.335 [1.275, 1.397]	< 0.001
M								
M0	1		1		1		1	
M1	1.776 [1.728, 1.825]	< 0.001	1.767 [1.718, 1.819]	< 0.001	1.902 [1.847, 1.957]	< 0.001	1.874 [1.818, 1.931]	< 0.001
Surgery								
No	1		1		1		1	
Yes	0.337 [0.311, 0.365]	< 0.001	0.510 [0.470, 0.554]	< 0.001	0.307 [0.281, 0.335]	< 0.001	0.492 [0.449, 0.539]	< 0.001
Chemotherapy								
No/Unknown	1		1		1		1	
Yes	0.438 [0.425, 0.452]	< 0.001	0.375 [0.363, 0.387]	< 0.001	0.442 [0.428, 0.457]	< 0.001	0.371 [0.359, 0.383]	< 0.001
Radiotherapy								
No/Unknown	1		1		1		1	
Yes	0.674 [0.658, 0.691]	< 0.001	0.658 [0.642, 0.675]	< 0.001	0.676 [0.659, 0.693]	< 0.001	0.662 [0.645, 0.679]	< 0.001
Median household income								
< 35,000	1				1			
> 75,000	0.957 [0.891, 1.028]	0.229			0.964 [0.895, 1.039]	0.334		
35,000–54,999	1.031 [0.961, 1.106]	0.397			1.038 [0.965, 1.116]	0.317		
55,000–74,999	0.994 [0.928, 1.066]	0.875			1.004 [0.934, 1.079]	0.921		
Marital status								
Divorced/Separated	1				1			
Married	0.982 [0.949, 1.016]	0.305			0.986 [0.951, 1.022]	0.434		
Single	0.969 [0.926, 1.013]	0.16			0.959 [0.916, 1.004]	0.074		
Widowed	1.040 [0.998, 1.084]	0.065			1.016 [0.973, 1.061]	0.463		

### Subgroup analysis

**Figure 5:**
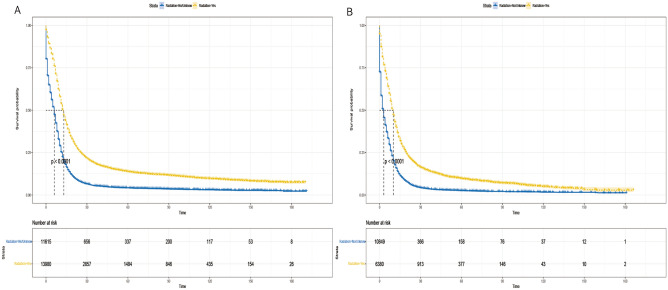
Kaplan–Meier survival curves for CSS in patients of different age in radiotherapy and non-radiotherapy groups. (**A**) age < 70, (**B**) age ≥ 70.

**Figure 6:**
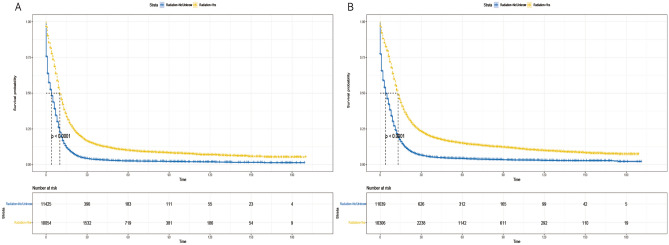
Kaplan–Meier survival curves of CSS in patients in radiotherapy and non-radiotherapy groups with different gender. (**A**) male, (**B**) female.

**Figure 7:**
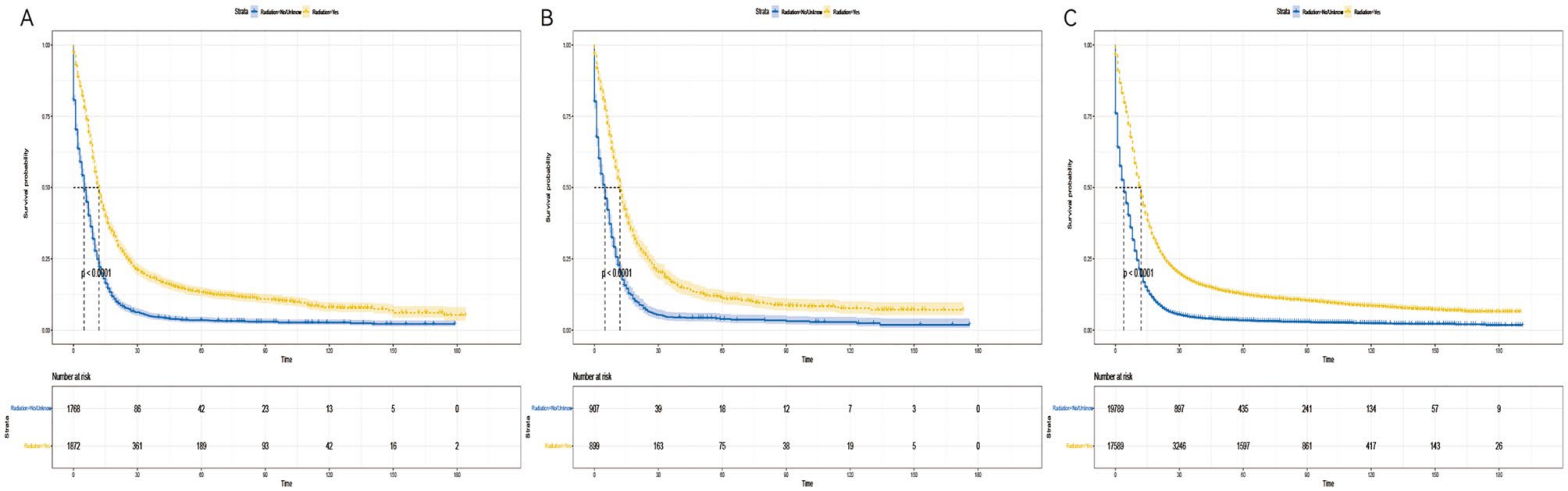
Kaplan–Meier survival curves for CSS in patients with different ethnic radiation groups and non-radiation groups. (**A**) Black, (**B**) other, (**C**) white.

**Figure 8:**
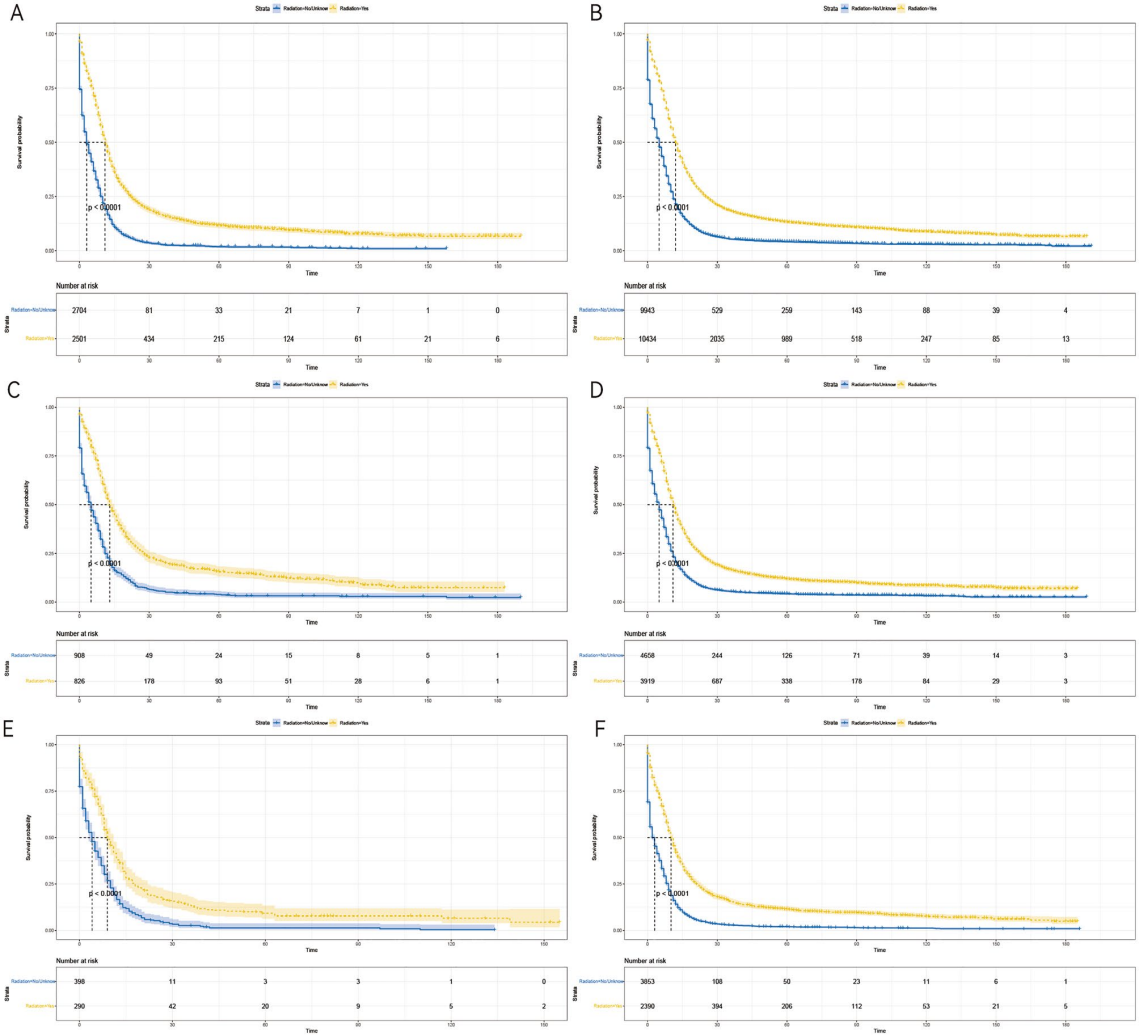
Kaplan–Meier survival curves of CSS in patients in the radiotherapy and non-radiotherapy groups with different primary tumor sites. (**A**) Main bronchus, (**B**) upper lobe of lung, (**C**) middle lobe of lung, (**D**) lower lobe of lung, (**E**) Overlapping lesion of lung, (**F**) unknown primary site.

**Figure 9:**
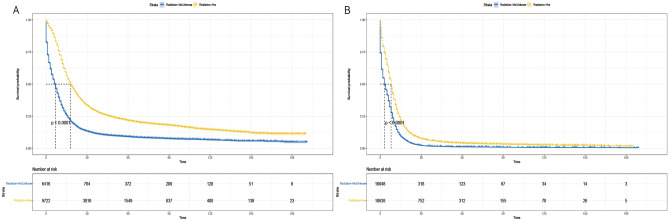
Kaplan–Meier survival curves of CSS in patients with in the radiotherapy and non-radiotherapy groups with different M stages. (**A**) M0, (**B**) M1.

**Figure 10:**
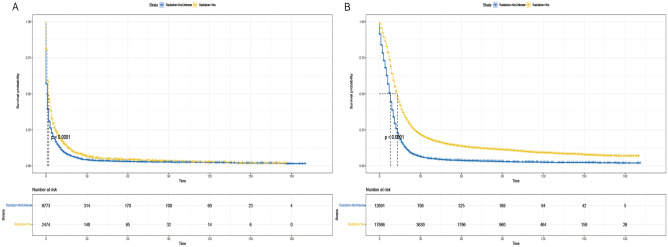
Kaplan–Meier survival curves of CSS of patients in the radiotherapy and non-radiotherapy groups with different treatment modalities. (**A**) with chemotherapy, and (**B**) without chemotherapy.

**Figure 11:**
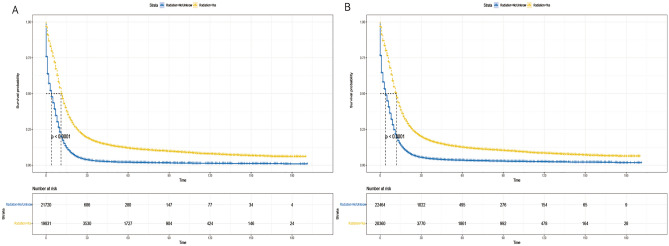
Kaplan–Meier survival curves of CSS in patients with radiotherapy and non-radiotherapy groups with different treatment modalities. (**A**) with surgery, and (**B**) without surgery.

Given the imbalance in the distribution of certain variables between the two groups in the matched dataset and the outcomes of the multivariate analysis, we conducted a subgroup analysis. We stratified a total of 42,824 patients based on identified variables, including age (< 70 and ≥ 70 years), gender (male and female), race (White, Black, Other), primary tumor site (main bronchus, upper lobe, middle lobe, lower lung, overlapping lesions, unknown primary site), M stage (M0, M1), chemotherapy (yes and no/unknown), and surgery (yes and no). Our findings revealed that the radiotherapy group exhibited a significant survival advantage across all subgroups defined by age (Fig. 5A,B), gender (Fig. 6A,B), race (Fig. 7A–C), primary tumor site (Fig. 8A–F), M stage (Fig. 9A,B), chemotherapy (Fig. 10A,B), and surgery (Fig. 11A,B) (all *P* < 0.0001).

## Discussion

Small Cell Lung Cancer (SCLC) is a high-grade neuroendocrine cancer characterized by its aggressive invasion, high metastatic potential, rapid progression, and dismal prognosis^13^. Consequently, managing SCLC poses significant challenges in clinical practice. Over 70% of SCLC patients are diagnosed at an advanced stage, resulting in limited survival prospects and rendering it an incurable disease, with treatment primarily focused on palliative care to extend survival duration^14^. The primary modalities for treating SCLC include surgery, radiotherapy, chemotherapy and so on. Radiotherapy has emerged as a crucial component in SCLC management, offering promising outcomes. Combining radiotherapy with chemotherapy has been shown to reduce the risk of mortality by 14% and elevate the 3-year overall survival rate by 5.4 ± 1.4% compared to chemotherapy alone^15^. Moreover, radiotherapy demonstrates efficacy in eliminating extensive residual tumors^16^. Therefore, this study aims to comprehensively assess independent risk factors for SCLC utilizing the extensive SEER database, examine survival disparities between radiotherapy and non-radiotherapy cohorts, and further investigate the survival impact of radiotherapy across various subgroups defined by risk factors.

Previous research has investigated the impact of radiotherapy on the prognosis of patients with localized SCLC. A meta-analysis revealed that thoracic radiotherapy significantly reduced mortality in localized patients by 14% (*P* = 0.001), correlating with a 5% increase in 3-year survival rates^17^. However, the effectiveness of radiotherapy in improving survival for patients with extensive-stage disease has been a topic of debate over the past decades, primarily due to conflicting findings regarding its efficacy in controlling intrathoracic tumors and managing the widespread metastatic nature of the disease. Early studies suggested a beneficial role for radiotherapy in controlling extensive-stage SCLC^18^. Nonetheless, subsequent investigations have yielded contradictory results^19–21^. It wasn’t until 1999 that Jeremic et al. highlighted the indispensable role of thoracic radiotherapy (TRT) in treating Extensive Disease Small Cell Lung Cancer (ED-SCLC)^22^. Subsequent studies have further substantiated this assertion. A retrospective study from the USA showed that the 1-year, 2-year, and median OS of ED-SCLC patients who received radiation were 27.8%, 9.3%, and 8 months and were significantly better than ED-SCLC patients who did not receive radiation (16.2%, 3.8%, and 4 months respectively; *P* < 0.0001)^23^. The conflicting findings observed in earlier studies could be attributed to the limitations of early diagnostic and staging methodologies, as well as outdated treatment protocols for radiotherapy and chemotherapy (e.g., 2D RT regimen, suboptimal RT dosage, and non-platinum-based chemotherapy). In this study, we employed PSM to control for confounding factors and achieve a balance in covariate differences between groups. Post-matched Kaplan–Meier analysis demonstrated that radiotherapy significantly improved OS and CSS in patients with SCLC. To estimate cancer-specific mortality accurately, we conducted a competing risk analysis. The results revealed that the non-radiotherapy group had significantly higher cancer-specific mortality compared to the radiotherapy group. Additionally, multivariate Cox analysis confirmed that radiotherapy was an independent prognostic factor in SCLC patients, with a strong positive correlation to both OS and CSS. Furthermore, subgroup analysis indicated that radiotherapy improved survival outcomes in both limited-stage and extensive-stage SCLC. These findings collectively suggest that radiotherapy plays a crucial role in extending survival and reducing the risk of cancer-related death for SCLC patients.

Moreover, our multivariate Cox analysis found that age, gender, race, primary tumor site, T stage, N stage, and, M stage, chemotherapy, surgery were also considered as important predictors of prognosis in SCLC. Our findings indicate that individuals aged 70 and above exhibit poorer OS and CSS. Consistent with previous research, older patients aged over 70 years tend to experience inferior OS compared to their younger counterparts, with observed median OS durations of 17.8 months and 23.5 months for older and younger patients, respectively^24^. Factors contributing to this disparity include declining physical health status and a higher prevalence of comorbidities among the elderly population. Additionally, older patients often present with lower stage completeness and receive less intensive chemoradiation and PCI^25^. These factors collectively contribute to the association between advanced age and poorer CSS outcomes. Our investigation revealed that gender serves as a significant prognostic factor, with females exhibiting improved OS and CSS. Previous studies found that CSS outperformed male females (HR 0.815, 95% CI 0.749–0.887, *P* < 0.001)^26^. Furthermore, an observational study conducted by Moser et al. reported that women had significantly better OS (29.6 months vs. 21.5 months, *P* = 0.03)^27^. Subsequently, a study involving over 1700 patients in 2010 corroborated these findings, showing a slight survival advantage for women over men (HR 0.88, 95% CI 0.79–0.99, *P* = 0.04)^28^. This observed survival advantage in females is attributed in part to the protective effects of estrogen, which is associated with longer life expectancy in women across various species, thereby contributing to higher OS and CSS outcomes in female patients^29^. Furthermore, our Cox analysis revealed that receiving chemotherapy and undergoing surgery were associated with improved OS and CSS among patients. Chemotherapy remains the cornerstone of both first- and second-line treatments for SCLC^30^. A review by Elegbede et al. of 404 SCLC patients treated at a tertiary Canadian cancer center from 2010 to 2016 demonstrated that chemotherapy significantly enhanced survival outcomes, particularly in extensive-stage disease (HR 0.33, 95% CI 0.22–0.48, *P* < 0.01). Additionally, patients receiving both chemotherapy and surgery experienced prolonged OS compared to those receiving chemotherapy alone (13 months vs. 9 months), with surgery also proving beneficial for OS among limited-stage SCLC patients (40 months vs. 8 months)^31^. Although surgery is traditionally considered appropriate for early-stage SCLC, recent studies have shown its potential benefits even in stage III N2 disease, particularly when followed by adjuvant chemotherapy or chemoradiotherapy^32^. This perspective was further supported by a retrospective analysis utilizing the SEER database, which reported superior OS and CSS outcomes in the surgical cohort compared to the non-operative group^33^.

Subgroup analysis revealed that the radiotherapy group demonstrated a significant survival advantage across various demographic and clinical characteristics, including patient age, sex, race, tumor site, chemotherapy, and surgery. In patients with SCLC, the elderly constitute a significant proportion, with approximately 50% of those with localized lung cancer being over 70 years old^16^. Addressing the optimal treatment approach for older individuals with SCLC has been a longstanding research focus. Given the decreased physical resilience and diminished drug tolerance observed in the elderly^25^, treatment decisions must be made judiciously. Radiotherapy emerges as a valuable adjunctive treatment modality in this context. Christopher Corso et al.^34^ demonstrated that the combination of radiotherapy with chemotherapy significantly improved OS in elderly SCLC patients, yielding a 3-year OS absolute benefit of 15.7%. Our study corroborates these findings by showing that radiotherapy confers survival benefits even among individuals aged over 70 years. Consequently, the primary treatment approach in elderly patients should involve a combination of chemotherapy and radiotherapy, offering greater additional OS advantages compared to chemotherapy alone^34^. Additionally, subgroup analysis revealed that the combination of radiotherapy with surgery or chemotherapy also led to improved survival outcomes. This is similar to the findings of the previous studies^35–38^. Kanaji et al. In a retrospective analysis included 366 SCLC patients receiving chemotherapy or chemoradiotherapy, and found that in LS-SCLC patients with idiopathic pulmonary fibrosis (IPF), chemoradiotherapy was associated with better progression-free survival (PFS) (281 days vs. 146 days, *P* = 0.0471) and OS (1163 vs. 355 days, *P* = 0.0012) compared to chemotherapy alone^35^. Two meta-analyses also supported the efficacy of adding thoracic radiotherapy (RT) to chemotherapy (CT) in improving survival among LS-SCLC patients^36,37^. Furthermore, Perry et al. showed that incorporating radiotherapy for primary tumors alongside combination chemotherapy significantly enhanced the complete response rate (*P* = 0.0013) and overall survival (*P* = 0.0099)^38^. In summary, our study suggests that radiotherapy may serve as a broadly effective treatment option for SCLC patients, further emphasizing its critical role in SCLC management.

However, this study has several limitations. Firstly, the presence of chronic diseases among patients, such as chronic obstructive pulmonary disease (COPD) and diabetes mellitus, along with Performance Status (PS), are significant prognostic factors for lung cancer patients undergoing radiotherapy. COPD, for instance, induces alterations in pretreatment lung parenchyma, resulting in a reduced lung area susceptible to radiotherapy, which could potentially impact treatment efficacy^39^. A study conducted in mainland China revealed that patients with hypertension and type 2 diabetes mellitus faced an elevated risk of mortality (HR 1.665, 95% CI 1.037–2.672; *P* = 0.00058)^40^, potentially diminishing the effectiveness of radiotherapy. Moreover, existing research suggests a correlation between poorer PS and lower 90-day survival rates post-radiotherapy^41^. Regrettably, the absence of this data in our database led to an underestimation of the survival rate, thereby introducing associated bias. Second, the completeness of evidence in radiation-related studies is flawed because the dose and range of RT cannot be obtained in the SEER database, and the radiation dose is a key factor affecting tumor control and tissue toxicity after radiotherapy, which plays an important role in patient prognosis. A retrospective analysis conducted using the NCDB revealed that a total radiation therapy (TRT) dose of 45 Gy yielded superior survival outcomes compared to TRT doses below 45 Gy (HR 0.78, *P* < 0.001)^42^. Similarly, another retrospective analysis demonstrated that a time-adjusted Biologically Effective Dose (tBED) of 50 Gy correlated with enhanced survival and disease control relative to tBED doses exceeding 50 Gy^43^. Nevertheless, for patients exhibiting good physical performance status and a longer estimated survival time, administering a higher TRT dose may result in improved local control and overall survival for extensive-stage small cell lung cancer (ES-SCLC)^44^. So optimal radiotherapy dose is an important issue for further discussion and we hope that more studies will focus on this. Finally, despite this study’s efforts to correct for suspected confounders using big data, the limitations inherent in retrospective studies could not be entirely eliminated. Therefore, we advocate for large-scale prospective studies to validate the conclusions drawn in this paper.

## Conclusion

Based on the large SEER database, this study concluded that radiotherapy could improve both OS and CSS in patients with SCLC. Age, sex, race, primary tumor site, T stage, N stage, M stage, chemotherapy, and surgery were also considered as important predictors of outcome in SCLC. Patients with SCLC may benefit from radiotherapy regardless of age, sex, race, primary tumor site, M stage, chemotherapy, and surgery.

## Data Availability

The data used in this study are available free of charge online at http://www.seer.cancer.gov on request.
